# Transforming the landscape of early cancer detection using blood tests—Commentary on current methodologies and future prospects

**DOI:** 10.1038/s41416-020-01223-7

**Published:** 2021-02-09

**Authors:** Minetta C. Liu

**Affiliations:** Division of Medical Oncology, Department of Oncology, Mayo Clinic, Rochester, MN USA

**Keywords:** Cancer genomics, Cancer screening

## Abstract

Early cancer detection should lead to an overall stage shift, less-intensive treatments and better patient outcomes. Current recommended screening programmes are limited to a handful of individual cancers. A multi-cancer early detection test that simultaneously detects and localises multiple cancers could reduce the morbidity and mortality associated with cancer.

## Main

A paradigm shift is underway from early detection of individual cancers to simultaneous early detection of multiple cancers with a single blood draw. This is possible through analysing circulating tumour cell-free DNA (cfDNA) and leveraging genomic technologies and machine learning, ranging from logistic regression to neural networks, to simultaneously detect multiple cancers and accurately predict the tissue of origin (TOO).^1,2^ Such a multi-cancer early detection (MCED) test would complement existing screening approaches to increase the absolute number of cancers detected (i.e. yield), including those lacking recommended screening programmes. However, for effective large-scale population screening, an MCED test should balance sensitivity and specificity, and be able to predict TOO,^1^ while minimising testing-associated potential risks, including overdiagnosis. Clinically relevant positive predictive value is essential,^1^ as the goal is to facilitate early intervention. A single test to screen for multiple cancers allows aggregation of prevalence rates, which allows such an MCED test—with a very low false positive rate (FPR) and moderate sensitivity across a broad range of cancer types—to detect many cancers that otherwise would go undetected until later stages when signs or symptoms are more likely to appear.^3^

### Development of an MCED test

Simultaneous detection of multiple cancers using cfDNA should include provisions for tumour localisation to guide diagnostic evaluation. The Circulating Cell-free Genome Atlas (CCGA) Study (NCT02889978) is a prospective, case-controlled study with longitudinal follow-up designed for discovery and validation of such a blood test. Biospecimens were collected from patients with newly diagnosed cancer before therapeutic intervention, and from healthy controls. Assessment of three prototype assays (whole-genome sequencing, targeted sequencing with a 507-gene panel, and whole-genome bisulphite sequencing) in the first substudy led to a focus on methylation for further assay development. More than 100,000 key methylation regions were selected as containing the most informative cancer-/tissue-specific methylation patterns versus noncancer controls. A robust targeted methylation assay for plasma cfDNA fragments was designed to detect and localise multiple cancers across stages with high specificity, as appropriate for a population-based screening programme.^1^ To assess performance of the machine-learning classifier for detection of >50 cancers and TOO prediction in the second CCGA substudy, samples from 6689 participants (2482 cancer, 4207 noncancer) were divided into training and validation sets.^1^ In the validation set, specificity was 99.3% (FPR, 0.7%).^1^ Stage I–IV sensitivity was 76.4% in a prespecified subset of 12 high-signal cancers (anus, bladder, colon/rectum, oesophagus, head and neck, liver/bile-duct, lung, lymphoma, ovary, pancreas, plasma cell neoplasm, stomach) and 54.9% in all cancers. Sensitivity increased with increasing disease stage: in prespecified cancers, from 39% in stage I to 92% in stage IV; in all cancers, from 18% in stage I to 93% in stage IV. TOO was predicted in 96% of samples with a cancer-like signal, of which 93% were accurate.^1^ This study demonstrated that informative methylation patterns can be leveraged for early multi-cancer detection, including detection of lethal cancers without recommended screening programmes.

Study limitations were noted. Stage and cancer distribution probably affected aggregate sensitivities. The FPR may have been overestimated because complete 1-year follow-up to confirm noncancer status was not available for all noncancer participants. Additionally, analyses are ongoing to improve TOO prediction accuracy. These issues will be addressed through two ongoing studies in asymptomatic intended-use screening populations: STRIVE (NCT03085888), a case-cohort study of ~100,000 women undergoing mammography screening; and SUMMIT (NCT03934866), a study of ~25,000 smokers and former smokers at high risk of lung cancer. PATHFINDER (NCT04241796), a prospective interventional study of ~6200 participants, will evaluate the clinical implementation of MCED testing by returning test results to clinicians and participants, ascertaining participant-reported outcomes and perceptions of the test and tracking diagnostic pathways toward resolution of a signal-detected test result.

### Landscape of MCED blood tests in development

**Fig. 1:**
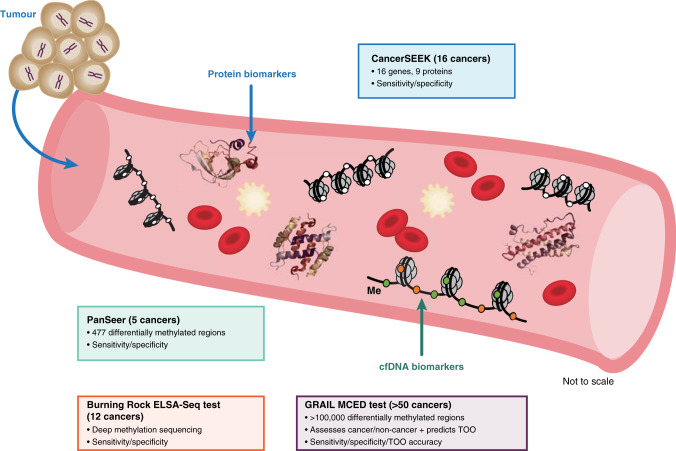
Attributes of multi-cancer detection methods. MCED multi-cancer early detection, TOO tumour of origin.

Other blood-based MCED tests are in development, yet standardised criteria have not been developed for clinical validity, benefit-risk, and clinical utility relative to MCED.^2,4^ Study comparisons are challenging because of differences in participant eligibility, cancers targeted for detection, methodology and performance metrics variability. For example, alternative approaches have been described that simultaneously detected 16 cancers based on analysis of tumour-specific mutations in cfDNA combined with protein biomarkers (CancerSEEK [Thrive Earlier Detection, Cambridge, MA, USA]), as well as 5 cancers (PanSeer [Singlera Genomics Inc, La Jolla, CA, USA]) or 12 cancers (Burning Rock, Guangzhou, Guangdong, China]) based on DNA methylation signatures^2,5–8^ (Fig. 1). Overall, these methods report high specificity (≥95%) with sensitivities of 16–100%, depending on cancer type and stage distribution. Limitations may include small prediagnosis sample size, cohorts that may not reflect the general population and absence of inherent TOO prediction.^5–8^

### Role of MCED tests in population-based cancer screening

MCED tests should be intended to complement recommended screening approaches by identifying cancers they do not cover. To screen for cancers with low occurrence in the general population, many people must be screened to detect a single cancer. Screening for all cancer types simultaneously would allow the prevalence of rare and common cancers to be combined, thus decreasing the number needed to screen. Additionally, combining high specificity and aggregate tumour prevalence is expected to result in positive predictive values exceeding those seen with single-cancer screening approaches that rely on high sensitivity but lower specificity.^3^ There is also an inverse relationship between aggregate sensitivity and absolute number of cancer cases detected; an MCED test with moderate sensitivity that detects nearly all cancer types is expected to maximise the number of detected cancer cases versus a test that artificially increases sensitivity by excluding cancers with low detection.^1^ Given the large absolute number of cancers that may be detected with an MCED test, an aggregate prevalence and high specificity (i.e. a very low FPR), combined with the ability to detect nearly any cancer type, may be the most effective approach.

Collectively, an opportunity exists for MCED testing to reduce cancer mortality and treatment costs, improve the effectiveness of cancer screening and complement single-cancer screening approaches. We are optimistic that findings from additional trials in intended-use screening populations will identify proper workflows for test implementation and facilitate adoption of MCED tests in clinical practice.

## Data Availability

Not applicable
